# Medication Beliefs Regarding P2Y12 Inhibitors: An Exploratory Thematic and Semantic Analysis to Better Understand the Key Barriers to Medication Adherence

**DOI:** 10.7759/cureus.85924

**Published:** 2025-06-13

**Authors:** Jin Sol G Lee, Yong Choi, Kyle Tejuco, Franklin Heng, Manoj Kesarwani

**Affiliations:** 1 Department of Internal Medicine, Harbor-UCLA (University of California Los Angeles) Medical Center, Torrance, USA; 2 Department of Internal Medicine, David Geffen School of Medicine at UCLA (University of California Los Angeles), Los Angeles, USA; 3 Department of Internal Medicine, Cedars-Sinai Medical Center, Los Angeles, USA; 4 Department of Health Information Management, School of Health and Rehabilitation Sciences, University of Pittsburgh Medical Center, Pittsburgh, USA; 5 College of Clinical Nutrition, UC (University of California) Davis, Davis, USA; 6 Department of Computer Science, UC (University of California) Berkeley, Berkeley, USA; 7 Department of Internal Medicine, Division of Cardiovascular Medicine, School of Medicine, UC (University of California) Davis, Davis, USA

**Keywords:** adherence, antiplatelet, platelet inhibitors, social media, x (formerly twitter)

## Abstract

Background

Nonadherence to P2Y12 inhibitor therapy following percutaneous coronary intervention (PCI) is associated with an increased risk of major adverse cardiovascular events. However, there are a variety of factors - intentional and non-intentional - that contribute to P2Y12 inhibitor nonadherence and result in poor patient outcomes.

Objective

The primary aim of this study was to identify natural, lived experiences of individuals taking P2Y12 inhibitors in the real world, using a widely used social media platform to identify common barriers to medication adherence.

Methods

Using deidentified data extracted from Twitter (now known as X) from January 1, 2016, to December 31, 2019, user comments pertaining to generic and brand names of P2Y12 inhibitor use were extracted using the GetOldTweets API (Application Programming Interface) with Python. Non-English tweets, duplicates, posts from automated bots, and those from users with clinical titles were excluded. The remaining posts were then manually reviewed based on the following inclusion criteria: (1) discussed treatment with P2Y12 inhibitors, (2) reflected personal experiences with P2Y12 inhibitors (e.g., patient or caregiver), and (3) provided sufficient context for qualitative analysis. A total of 366 eligible posts were analyzed using conventional content analysis and grouped into five key themes, which were further categorized into positive, negative, or neutral sentiments.

Results

Five representative themes emerged from the data, including adverse drug reactions (137 or 37%), affordability (93 or 25%), inconvenience of taking the medication (77 or 21%), patient-perceived response to treatment (42 or 12%), and provider trust and communication (17 or 5%). Most posts (205 or 56%) expressed negative sentiments (e.g., anger, frustration, sadness, fear) about taking P2Y12 inhibitors, while 26 (7%) shared positive sentiments (e.g., joy, gratitude, happiness, excitement). The remaining 135 (37%) were classified as neutral.

Conclusions

This study, using natural, lived experiences of users with P2Y12 inhibitors, highlights the complex factors that may contribute to nonadherence to this medication. By better understanding patient attitudes and beliefs regarding this class of medication, as expressed in user comments, barriers to adherence can be identified and form the basis for future studies aimed at developing meaningful interventions to ensure consistent P2Y12 inhibitor use among post-PCI patients.

## Introduction

P2Y12 inhibitors are an essential component of goal-directed medical therapy in patients with acute and chronic coronary syndromes that require percutaneous coronary intervention (PCI). Used in combination with aspirin, P2Y12 inhibitors form the basis of dual antiplatelet therapy (DAPT) and have been shown to reduce the risk of major adverse cardiovascular events, including stent thrombosis, myocardial infarction, and death [1,2]. However, approximately 40% of patients are still nonadherent to P2Y12 inhibitor therapy at 12 months, and - more concerningly - 15% to 20% fail to fill their prescriptions within 30 days of hospital discharge [1,3].

Current European and U.S. guidelines provide a Class I recommendation for a ≥6-month course of DAPT in patients with stable angina pectoris undergoing PCI. Among the population with acute coronary syndrome (ACS), the same guidelines indicate at least a ≥12-month treatment period of DAPT after PCI [2]. Although previous studies suggest cardiovascular benefit in DAPT courses well beyond 12 months among post-PCI patients at high ischemic risk, more recent randomized controlled trials have indicated that shorter courses of DAPT - one to three months - may be a safe alternative among both ACS and non-ACS patients [4]. Abbreviated courses of DAPT (one to three months), followed by antiplatelet monotherapy - either low-dose aspirin or a P2Y12 inhibitor - are also a reasonable option in patients at high bleeding risk, regardless of lesion complexity. While major bleeding is due to a variety of factors (e.g., vascular access site and patient comorbidities), strategies to prevent it, such as shorter durations of DAPT, are critical to ensure optimal PCI outcomes. 

Prior interventions have tried to address nonadherence through education [5,6], cost coverage [7], and reminder technologies [8]. However, these strategies have yielded only marginal improvements in adherence. Establishing medication adherence requires multifactorial decision-making, driven fundamentally by patient beliefs and attitudes, and facilitated by conversations between the patient and provider. Prior qualitative research has used surveys, interviews, and focus groups to better assess adherence [9-12], but is limited by small sample size and the potential for social desirability bias. Social media, particularly Twitter, with over 200 million active users and 400 million daily posts, provides a rich, large dataset for understanding patients’ lived experience and their health-related beliefs [13]. Prior research has successfully utilized Twitter to explore attitudes toward cardiovascular medications like statins [14].

In this study, we analyzed Twitter posts regarding their P2Y12 inhibitor medications to identify patient beliefs and attitudes, aiming to explore barriers and facilitators to effective patient adherence.

## Materials and methods

This study adhered to the relevant aspects of the Consolidated Criteria for Reporting Qualitative Research (COREQ) reporting guideline [15]. All data collected in this study were publicly available, de-identified data extracted from Twitter according to their terms of use. Thus, approval from the IRB was not required, as the data were publicly available and this was not human subjects research. Extracted data were further stored, managed, and represented according to the Association of Internet Researchers (AOIR) guidelines for ethical internet research [16].

Selection criteria

We used search terms including generic and brand names of P2Y12 inhibitors (clopidogrel, Plavix; ticagrelor, Brilinta; prasugrel, Effient) to collect tweets from January 1, 2016, to December 31, 2019, using the GetOldTweets API with Python [17]. To avoid excluding potentially relevant posts, we did not limit the dataset by medical terms (e.g., heart attack), and instead, three authors (JL, YC, and KT) manually reviewed each post for relevance. However, for the purpose of feasibility of study design, aspirin-related posts were excluded due to their broad use beyond DAPT (e.g., headache and pain). Examples of posts excluded during the manual review are provided in Table 3 (see Appendix).

We excluded non-English language tweets, exact duplicates (e.g., retweets without additional comment), posts from users with overtly clinical titles in their profiles (e.g., "cardiologist," "MD" to focus on patient/caregiver perspectives), and posts identified as originating from automated bots. Bot detection was conducted manually by two researchers (e.g., JL and YC) based on predefined criteria, including a high volume of repetitive or spam-like content, excessive use of external links to commercial or unrelated websites, overt advertising language, and a lack of human-like conversational engagement. Posts flagged as potential bots by one researcher were independently reviewed and confirmed by the second reviewer. 

**Figure 1:**
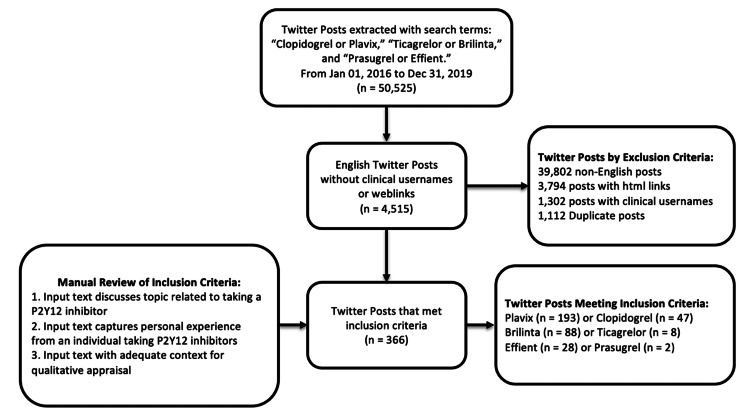
Flow diagram of inclusion and exclusion of Twitter posts

Following these exclusions, posts were then manually reviewed by three authors (JL, YC, and KT) for the following inclusion criteria: (1) the post explicitly discussed treatment with one or more P2Y12 inhibitors; (2) the content reflected a personal experience, belief, or attitude regarding P2Y12 inhibitors from the perspective of a patient or caregiver; and (3) the post provided sufficient context for qualitative analysis (Figure 1). 

Qualitative appraisal

Our qualitative analysis was guided by a constructionist approach, recognizing that individuals actively construct meaning from their experiences, particularly when articulating subjective beliefs and attitudes on social media like Twitter. This lens was chosen to understand how patients and caregivers make sense of and share their lived experiences with P2Y12 inhibitors, acknowledging our role in interpreting these constructed narratives. For the analysis itself, we conducted Braun and Clarke’s six-phase guide for thematic analysis [18]. This method was selected for its flexibility and suitability for identifying, analyzing, and reporting patterns (themes) within qualitative data, aligning well with our constructionist orientation and our goal of exploring subjective experiences. The first author (JL) conducted an initial pass, annotating tweets in Excel (Microsoft® Corp., Redmond, WA, USA), to identify preliminary themes using conventional content analysis. Each post was also categorized as positive, negative, or neutral in sentiment, based on the overall tone and explicit content of the tweet. Following this initial work, the matrix analysis using the Excel file was performed by JL, YC, and MK, upon which a consensus review was achieved through triangulation of data. Minimal discrepancies were found between the reviewers, and consensus was reached on sentiment classifications for each post to ensure consistent interpretation across the dataset.

## Results

Of the total 366 posts that met inclusion criteria, 276 (75%) were written by individuals taking P2Y12 inhibitors, while 90 (25%) were from caregivers or others close to the patient. Most posts, 205 (56%), expressed negative sentiments (e.g., anger, frustration, sadness, and fear) about taking P2Y12 inhibitors, while 26 (7%) shared positive sentiments (e.g., joy, gratitude, happiness, and excitement). The remaining 135 (37%) were classified as neutral. Five main themes emerged from the data: adverse drug reactions (137, or 37%), affordability (93, or 25%), inconvenience of taking the medication (77, or 21%), patient-perceived response to treatment (42, or 12%), and provider trust and communication (17, or 5%) (Tables 1-2).

**Table 1:** P2Y12 inhibitor CARRE concordance construct: number of health related tweets mentioning a P2Y12 inhibitor by theme and subtheme

Category of Theme: Subtheme	Description	No. of Tweets (%)
Provider Trust & Communication	These tweets contain statements that describe feelings of mistrust due to gaps in communication between the patient and provider.	17 (4.7%)
Affordability: High Drug Cost	These tweets contain posts discussing the high cost of the medication.	73 (20%)
Affordability: Lack of Insurance Coverage	These tweets contain posts discussing the individual's struggle to afford the medication due to a lack of insurance coverage.	20 (5.5%)
Response to Treatment: Belief in Treatment Effectiveness	These tweets contain opinions held by the individual on the efficacy of the P2Y12 inhibitor.	42 (11.5%)
Adverse Drug Reaction: Bleed or Bruise	These tweets contain the user’s experience of bleeding and nuisance bleeding (or bruising) from the medication.	88 (24%)
Adverse Drug Reaction: General Bad Feeling	These tweets contain the user’s experience of a general bad feeling from medication.	6 (1.6%)
Adverse Drug Reaction: Shortness of Breath	These tweets contain the user’s experience of shortness of breath, which is a known side effect from a specific P2Y12 inhibitor (ticagrelor/Brilinta).	43 (11.7%)
(E) Inconvenience: Refilling Medication	These tweets contain user’s experience with the inconvenience experienced from filling or refilling the medication.	17 (4.6%)
(E) Inconvenience: Restrictions on Lifestyle	These tweets contain user’s experience with the inconvenience experienced from the restrictions placed on the individual’s life by taking the medication.	60 (78%)

**Table 2:** Thematic analysis and representative quotes

Category of Theme: Subtheme	Representative Quotes	No. of Tweets (%)
Provider Trust & Communication	“Me too, but I trust my doctors to tell me what to take. They haven't let me down, except for one. Forced me to switch from Plavix to Brilinta (sp?) and nearly ruined my life. A real arrogant ass, he was. Found that Astrazenica was giving him some damned good bucks. I dumped him.” “Yup...thanks to my cardiologist, I need to take plavix for the rest of my life...even though I asked for bare metal stents, he placed drug-eluting stents…thanks for nothing buddy”.	17 (4.7%)
Affordability: High Drug Cost	“Did you say they charged you 400.00 for your Brilinta? I had heart attack in Nov 18. Ive been paying 5.00 a month BUT NOW somehow its 400.00 for the next 3 months. Its bullshit. I would have picked plavix.” “It is terrible! My mom is 93 and only income is her SSN. The cost of #brilinta even with a savings card is $400 per month! I am praying she is approved for assistance. 🙏🙏🙏” “[user's] choice was literally: pay rent or pay for plavix etc. She chose 2 keep the roof over her daughters' heads over her meds.”	73 (20%)
Affordability: Lack of Insurance Coverage	“I suffered my first heart attach on March 23, 2013 and my second on February 17, 2017. During this period of time I have been on Brilinta. Just recently I have switched jobs and my health insurance does not cover a majority of the cost.” “I'm a senior-had heart attack doc wants me on Brilinta for at least a yr, not Plavix. Just got letter I'm in donut hole-med$ will quadruple.” “Our #ACA deductible is so high my husband who is on #effient which costs us $348 a month doesn't take daily because we can't afford #Masa”	20 (5.5%)
Response to Treatment: Belief in Treatment Effectiveness	“I have 2 stents that I have to take Plavix for or they can close up and cause another heart attack!!! No meds=death sentence.” “This is a recipe for me to stay alive after heart surgery. The effient is anti-clotting agent and the aspirin is a blood thinner.” “Plavix doesn't work on > 40% of the South Asian population bc we don't produce the enzyme required to synthesize it into our systems. Most doctors don't know and prescribe anyway. need a genetic test to verify it doesnt work otherwise insurance won't cover alternative.”	42 (11.5%)
Adverse Drug Reaction: Bleed or Bruise	“Generic drugs almost killed me and I can not sue them under federal laws. Generic Plavix and Plavix gave me 2 brain bleeds. Lucky God savedme” “I’ve been on aspirin & ticagrelor since December last year. If you think my legs are bad, you should see my stomach & upper arms. I take injections every day for my diabetes, you’d think I’d went 12 rounds w/Anthony Joshua the amount of bruising & discolouration I have 🥴🥊 lol”	88 (24%)
Adverse Drug Reaction: General Bad Feeling	“I do not need the Lipitor, and I want to get off Lopresser and Brilinta. They all make me numb and weird feeling sometimes.” “@AstraZeneca Is there any way I can counteract side effects from #Brilinta, like tiredness and dizziness? They're starting to really get to me. Thank you.”	6 (1.6%)
Adverse Drug Reaction: Shortness of Breath	“One of the meds they have my dad on is brilinta. Its side effects are chronic nose bleeds and shortness of breathe. So when you wake up fr om a nap or overnight it feel like your being suffocated. But if you can ignore that it really helps your #heart. 🙄🙄😶 #awful”	43 (11.7%)
(E) Inconvenience: Refilling Medication	“Why is it so difficult to fill a prescription? I need a refill of my prasugrel, and I told @Walgreensthat I would only accept prasugrel from @PrascoAGs. So they filled the Rx with another generic. Not good enough! They claim @PrascoAGs is out of it until the end of June! :-( “What if I'm out of refills and also non-compliant? I want my Plavix now even though I've been out for six weeks. I might die!”	17 (4.6%)
(E) Inconvenience: Restrictions on Lifestyle	“Guess I have to quit drinking with the Plavix the Dr. gave me! Damn!” “Urgent prayer request! My dad needs another bladder cancer operation, but the cardiologist does not want it to take place until he's been on plavix for a year, but he's losing alot of blood in his urine. Please pray that the bleeding stops. Thank you for your prayer's. 🌹👏❤🙏”.	60 (78%)

Adverse drug reactions

Of the total 137 posts, 86 (63%) were negative, 51 (37%) neutral, and none were positive. Bruising/bleeding (88, or 24%) was the most reported side effect, followed by shortness of breath linked to Brilinta/ticagrelor (43, or 11.7%), and general bad feeling (6, or 1.6%). 

Affordability

Of the total 93 posts, 63 (68%) shared negative sentiment, expressing struggles with paying for the cost or finding insurance coverage for the medication. Those with neutral sentiment were seeking help from other Twitter users on how to navigate coverage for the medication (24, or 26%). Lastly, those with positive sentiment celebrated finding coverage for the medication (6, or 6%).

Inconvenience

Of the total 77 posts, 37 (49%) expressed lifestyle restrictions with taking P2Y12 inhibitors, 25 (32%) shared difficulty with taking the medication, and 17 (22%) shared challenges with obtaining and filling refills of their medications. 

Patient perceived efficacy

Of the total 42 posts, the majority were neutral (25, or 60%), sharing matter-of-fact statements explaining why they have to take the P2Y12 inhibitors. Only 10 (24%) expressed negative sentiment, sharing concerns about drug resistance, while seven (16%) posts expressed positive sentiment, celebrating successful treatment completion.

Provider trust and communication

Of the total 17 posts, the majority (13, or 76%) were negative, with feelings of judgment from their providers, mistrust of their providers, and big pharmaceutical companies.

## Discussion

Patient perspectives of P2Y12 inhibitors have previously been assessed using qualitative research through focus group interviews [9-12]. Our research shows that social media data can be used to further enrich our understanding of patient beliefs and attitudes about medications such as P2Y12 inhibitors. Themes discovered in existing literature were also found in our research, including systematic barriers (such as issues with affordability), medication-related barriers (such as adverse effects), and patient- and provider-level factors (such as establishing concordant beliefs about the medications). Interestingly, while prior qualitative studies did not represent each theme equally, our research included all themes from each individual study. Our study was able to capture a composite list of themes from multiple studies, highlighting the potential for large-scale Twitter data to provide a strong representation of qualitative data from the user perspective. 

We found that the adverse effects of the medication represented a large majority of posts related to P2Y12 inhibitors, highlighting the existing treatment burden associated with taking the medication itself - likely contributing to reduced motivation to remain adherent. Furthermore, patients are vulnerable to additional threats to their motivation through modifiable systematic barriers, including issues with affordability and inconveniences (e.g., difficulty getting refills). Thus, our research provides further contextual evidence to help bring awareness to - and humanize - these real-life challenges that patients struggle with daily by offering real examples of users’ natural lived experiences (Table 2). Strategies to reduce the treatment burden could include prescribing generics when possible, automating refills, and mail-delivering medications, as echoed in prior literature [19,20]. Furthermore, it will be important for all providers (e.g., inpatient and outpatient providers, generalists and specialists, pharmacists, and nurses) to recognize these vulnerabilities and leverage their critical roles to continuously educate and counsel patients on the importance of adherence throughout the care continuum.

For instance, we found that many users want to be more involved in their healthcare decisions. They find it important to know why they need to take their medications and, additionally, seek reassurance from their providers that the medication will work as intended. Moreover, trust between users and their providers is crucial to facilitate discussions that lead to concordant beliefs about the medication. Thus, our social media data may help inform effective communication strategies by making common beliefs and attitudes about P2Y12 inhibitors more well-known. For example, our findings support the need to remind providers to help patients improve their understanding of the treatment indication for the medication, as well as the need to discuss anticipated side effects with patients. Finally, social networks also play a key role, with a quarter of Twitter posts coming from caregivers or others close to patients, underscoring their influence on adherence.

Strengths and limitations

Twitter offers a new, unique platform for analyzing real-life health-related experiences without the social desirability bias that may occur in more traditional focus group interviews or surveys [13]. It also provides larger amounts of qualitative data than traditional forms of research and, thus, has the potential to uncover additional themes by aggregating more user perspectives. On the other hand, efficient analysis of social media data will need to leverage natural language processing (NLP) tools, which are currently limited in parsing complex social media language for content and sentiment analysis - especially as it relates to health-related information. Thus, at this time, reliable analysis still requires a decent amount of manual review, which can be time-intensive. Finally, our findings may not fully represent all patients, as Twitter users tend to be younger, more educated, and may express more extreme views than the general public - although these demographic characteristics are also changing [13]. As methods and tools like NLP continue to evolve for social media research, Twitter (X) remains a valuable source of qualitative data that can help inform efforts to better understand the complex set of beliefs and attitudes that drive users’ behavior around adherence.

## Conclusions

Our study underscores the complexity of factors affecting P2Y12 inhibitor adherence, based on analysis using real-world Twitter data. Key themes included communication about treatment efficacy, adverse drug reactions, affordability, and inconvenience, with most patients expressing negative experiences. By better understanding these patient-specific barriers, we hope to help provide a foundation for future interventions that could meaningfully improve adherence to these life-saving medications. 

## Appendices

**Table 3:** Examples of posts that were excluded during manual review

Inclusion Criteria	Example Tweet That Did Not Meet the Respective Inclusion Criteria
(1) input text captured a perspective of a primary or secondary user personally affected by issues related to taking P2Y12 inhibitors (e.g., patient or caregiver)	Doctors use this in emergency situations in the hospital, but transfer patients to Plavix, Xarelto, or Eliquis after a crisis.
(2) The input text had to discuss a topic related to taking one of the P2Y12 inhibitors	Primary Tweeter: VITC: 500 mg, ASPIRIN: 81 mg, OMEGA-3: 1 soft gel, CLOPIDOGREL: 75 mg, MULTIVITAMINS: 1 tablet. Off to work
(3) The input text had to have adequate context within the post to complete an appropriate qualitative appraisal using descriptive content and sentiment analysis	I use to bed at 9 pm wake up at 5:30 automatic. Now I need a pill. I take my plavix, lunesta & some trazadone & hopefully get solid 6 hrs.

## References

[1] Ho PM, Tsai TT, Wang TY (2010). Adverse events after stopping clopidogrel in post-acute coronary syndrome patients: insights from a large integrated healthcare delivery system. Circ Cardiovasc Qual Outcomes.

[2] Virani SS, Newby LK, Arnold SV (2023). 2023 AHA/ACC/ACCP/ASPC/NLA/PCNA guideline for the management of patients with chronic coronary disease: a report of the American Heart Association/American College of Cardiology joint committee on clinical practice guidelines. Circulation.

[3] Choi JM, Lee SH, Kang M, Choi JH (2020). Impact of medication adherence to dual antiplatelet therapy on the long-term outcome of drug-eluting or bare-metal stents. PLoS One.

[4] Carvalho PE, Gewehr DM, Nascimento BR (2024). Short term dual antiplatelet therapy after drug eluting stenting in patients with acute coronary syndromes: a systematic review and network meta-analysis. JAMA Cardiol.

[5] Ho PM, Lambert-Kerzner A, Carey EP (2014). Multifaceted intervention to improve medication adherence and secondary prevention measures after acute coronary syndrome hospital discharge: a randomized clinical trial. JAMA Intern Med.

[6] Choudhry NK, Isaac T, Lauffenburger JC (2018). Effect of a remotely delivered tailored multicomponent approach to enhance medication taking for patients with hyperlipidemia, hypertension, and diabetes: the STIC2IT cluster randomized clinical trial. JAMA Intern Med.

[7] Choudhry NK, Avorn J, Glynn RJ (2011). Full coverage for preventive medications after myocardial infarction. N Engl J Med.

[8] Choudhry NK, Krumme AA, Ercole PM (2017). Effect of reminder devices on medication adherence: the REMIND randomized clinical trial. JAMA Intern Med.

[9] Lambert-Kerzner A, Havranek EP, Plomondon ME (2015). Perspectives of patients on factors relating to adherence to post-acute coronary syndrome medical regimens. Patient Prefer Adherence.

[10] Decker C, Garavalia L, Garavalia B, Spertus JA (2008). Clopidogrel-taking behavior by drug-eluting stent patients: discontinuers versus continuers. Patient Prefer Adherence.

[11] Garavalia L, Ho PM, Garavalia B, Foody JM, Kruse H, Spertus JA, Decker C (2011). Clinician-patient discord: exploring differences in perspectives for discontinuing clopidogrel. Eur J Cardiovasc Nurs.

[12] Pettersen TR, Fridlund B, Bendz B, Nordrehaug JE, Rotevatn S, Schjøtt J, Norekvål TM (2018). Challenges adhering to a medication regimen following first-time percutaneous coronary intervention: a patient perspective. Int J Nurs Stud.

[13] McDonald L, Malcolm B, Ramagopalan S, Syrad H (2019). Real-world data and the patient perspective: the PROmise of social media?. BMC Med.

[14] Golder S, O'Connor K, Hennessy S, Gross R, Gonzalez-Hernandez G (2020). Assessment of beliefs and attitudes about statins posted on Twitter: a qualitative study. JAMA Netw Open.

[15] Tong A, Sainsbury P, Craig J (2007). Consolidated criteria for reporting qualitative research (COREQ): a 32-item checklist for interviews and focus groups. Int J Qual Health Care.

[16] Markham A, Buchanan E (2012). Ethical Decision-Making and Internet Research: Recommendations From the AoIR Ethics Working Committee (Version 2).

[17] Henrique J (2019). Jefferson-Henrique/GetOldTweets-Python. GetOldTweets-python.

[18] Braun V, Clarke V (2006). Using thematic analysis in psychology. Qual Res Psychol.

[19] Ferdinand KC, Senatore FF, Clayton-Jeter H (2017). Improving medication adherence in cardiometabolic disease: practical and regulatory implications. J Am Coll Cardiol.

[20] Kolandaivelu K, Leiden BB, O'Gara PT, Bhatt DL (2014). Non-adherence to cardiovascular medications. Eur Heart J.

